# Ocular Biometry in Primary Angle-Closure Glaucoma Associated with Retinitis Pigmentosa

**DOI:** 10.1155/2017/9164846

**Published:** 2017-12-31

**Authors:** Jiangang Xu, Zhikun Ouyang, Yangfan Yang, Xiaoyu Cai, Zhonghao Wang, Mingkai Lin, Xiulan Zhang, Xing Liu, Minbin Yu

**Affiliations:** State Key Laboratory of Ophthalmology, Zhongshan Ophthalmic Center, Sun Yat-sen University, Guangzhou 510060, China

## Abstract

**Background:**

Retinitis pigmentosa (RP) comprises a group of inherited disorders in which patients typically lose night vision in adolescence and then lose peripheral vision in young adulthood before eventually losing central vision later in life. A retrospective case-control study was performed to evaluate differences in ocular biometric parameters in primary angle-closure glaucoma (PACG) patients with and without concomitant RP to determine whether a relationship exists between PACG and RP.

**Methods:**

We used ultrasound biomicroscopy (UBM) to measure anterior chamber depth (ACD). A-scan biometry was carried out to measure lens thickness (LT) and axial length (AL). Propensity score matching and mixed linear regression model analysis were conducted. 23 patients with chronic primary angle-closure glaucoma (CPACG) associated with RP, 21 patients with acute primary angle-closure glaucoma (APACG) associated with RP, 270 patients with CPACG, and 269 patients with APACG were recruited for this study.

**Results:**

There were no significant differences on ACDs, ALs, and relative lens position (RLP) (*P* > 0.05) between patients with PACG associated with RP and patients with PACG; however, patients with APACG associated with RP had a significantly greater LT than patients with APACG (*P* < 0.05).

**Conclusion:**

Patients with PACG associated with RP had the same biometric parameter characteristic as the patients with CPACG and APACG. This may suggest that RP is a coincidental relationship with angle-closure glaucoma.

## 1. Introduction

Retinitis pigmentosa (RP) comprises a group of inherited disorders in which patients typically lose night vision in adolescence and then lose peripheral vision in young adulthood before eventually losing central vision later in life due to progressive rod and cone photoreceptor cell loss [1–3]. However, some extreme cases of the disease are characterized by a slow course that never leads to blindness or rapid deterioration that leads to blindness in less than two decades [4, 5]. The prevalence of RP in the US and Europe is approximately 1 : 3500 to 1 : 4000 [1]; however, the prevalence in China remains unknown. RP is not specific to a particular ethnic group and is believed to occur at similar frequencies in various populations; however, no data regarding its prevalence in other populations have been reported. Because the visual field is severely constricted in RP, most patients become legally blind at 40 years of age. In most typical RP cases, rod functional deterioration occurs faster than cone functional deterioration. In other types of RP, however, rod functional deterioration occurs in conjunction with cone functional deterioration [6]. In some rare cases of RP, visual acuity loss and defective color vision are the most obvious early symptoms of the disease because the rate at which affected patients lose cone sensitivity exceeds that at which they experience rod functional deterioration. These changes result in cone-rod dystrophy [1]. Additionally, slit-lamp biomicroscopy and ophthalmoscopy examination studies have demonstrated that approximately 50% of individuals with RP suffer from posterior subcapsular cataracts [1].

The cooccurrence of glaucoma and RP [7], although uncommon, has been observed by many ophthalmologists. Most reported cases have involved patients with open-angle glaucoma, especially in Caucasian populations [6, 8, 9]. We have noted patients with RP and angle-closure glaucoma more often in our works, which comprised Chinese populations, than other groups have in their studies. In a single report by Badeeb et al. [9], the prevalence of primary angle-closure glaucoma (PACG) and RP in patients over 40 years of age was 1.03%. In addition, Badeeb et al [9] believed that a strong association existed between angle-closure glaucoma and RP. The prevalence of PACG with RP in the Chinese population is higher than that in the Japanese population [10, 11]. However, the relationship between glaucoma and RP remains controversial. In this study, we focused on the biometric parameters of patients with both types of angle-closure glaucoma and RP and investigated the anatomical characteristics of these comorbid diseases.

## 2. Methods

### 2.1. Patients with PACG and RP

This was a retrospective, clinic-based study. From August 27, 2003, to May 3, 2016, 44 patients with PACG associated with RP, 270 patients with chronic primary angle-closure glaucoma (CPACG), and 269 patients with acute primary angle-closure glaucoma (APACG) were evaluated at the Zhongshan Ophthalmic Center and subsequently enrolled in the study. The investigation adhered to the guidelines of the Declaration of Helsinki, and the ethics committee approval was obtained from the Zhongshan Ophthalmic Center of Sun Yat-sen University in Guangzhou.

CPACG was diagnosed based on the presence of the following characteristics [12–14]: (1) a narrow synechial angle, (2) an IOP ≥ 22 mmHg, (3) glaucomatous optic disc damage leading to visual field loss in the presence of synechial angle closure characterized by the loss of two or more quarters of the circumference of the angle, and (4) a lack of acute ocular hypertension-induced ischemic damage in the anterior segment of the eye.

APACG was diagnosed based on the presence of the following characteristics [15–17]: (1) acute increases in IOP and angle closure; (2) acute ophthalmalgia, blurred vision, or nausea and vomiting; and (3) ischemic damage caused by acute ocular hypertension, as well as by ciliary or mixed injection, corneal edema, and glaucomatous flecks.

RP was diagnosed based on the presence of the following characteristics [1, 18–20]: (1) symptoms of nyctalopia; (2) pigmentary deposits resembling bone spicules originating from the peripheral retina, attenuation of the retinal vessels, waxy pallor of the optic disc, and various degrees of retinal atrophy, demonstrated by funduscopy; and (3) dramatic reductions in a-wave and b-wave amplitudes affecting the scotopic system (rods) to a greater extent than the photopic system (cones), demonstrated by electroretinography.

Patients presenting with features consistent with both PACG and RP were diagnosed with PACG associated with RP. All the patients with CPACG had vision in both eyes. The worst eye, as determined by visual field testing, was used in the study. Among patients with APACG, the acute-stage eye was used in the study. No patients exhibited evidence of fundus abnormalities other than those associated with RP or previous ophthalmic operations.

## 3. Ocular Biometric Assessment

All patients underwent full ocular examinations comprising visual acuity assessments; IOP (Goldmann applanation tonometer) measurements; slit-lamp biomicroscopy, gonioscopy, and fundoscopy; Humphrey visual field tests; and ultrasound biomicroscopy (UBM, performed in a dark room). Patients were examined in the supine position, and fixation and accommodation were kept constant throughout the examination. Radial scans were performed in the inferior, temporal, superior, and nasal quadrants of each eye, and the probe was held perpendicular to the ocular surface to measure the anterior chamber depth (ACD). A-scan biometry was used to measure the axial length (AL) and lens thickness (LT), and the following formula was used to measure the relative lens position (RLP):

(1) $$\begin{array}{c} RLP=\frac{ACD+\left(1/2\;LT\right)}{AL}. \end{array}$$

### 3.1. Statistical Analyses

SPSS 18.0 software (IBM SPSS, Armonk, NY, USA) was used for data analysis. R software, along with the corresponding version of SPSS software and the appropriate plug-ins, was used for propensity score matching, which was completed for both groups (CPACG associated with RP versus CPACG and APACG associated with RP versus APACG). The cases were matched separately in a 1 : 4 ratio. According to statistical analysis, when data ratio of the experimental group and the control group is 1 to 4, the statistical efficiency was largest. And propensity score matching was used to select the control groups from the recruited patients. In addition, the propensity score logic standard deviation tolerance (caliper) setting was 0.02, according to the nearest neighbor matching principle and the substitution principle (i.e., individual cases could not be selected multiple times). Patients with CPACG associated with RP were age- and gender-matched with the most similar patients with CPACG. Similarly, patients with APACG associated with RP were age- and gender-matched with the most similar patients with APACG. Qualitative variables were expressed as frequencies (constituent ratios), and between-group comparisons were performed using Fisher's exact tests. Quantitative variables were expressed as the mean ± standard deviation. *t*-tests were used to compare normal distributions, and Wilcoxon tests were used to compare abnormal distributions.

We used mixed linear regression to model the ocular biometric parameters for each individual enrolled in the study. A mixed-effects model facilitates explicit specifications of heterogeneity by assigning random-effects terms to parameters with effects that vary between individuals. A linear mixed-effects model was used to analyze the differences between the two groups and to control for the matching associations within each pair. The limit of statistical significance was set at *P* < 0.05.

## 4. Results

A total of 23 Chinese patients with CPACG associated with RP and 21 Chinese patients with APACG associated with RP were recruited for this study. In addition, 270 patients with CPACG and 269 patients with APACG were recruited for the study to serve as a control group. To account for the differences in gender and age between the two groups, we used propensity score matching (1 : 4) to compare the two groups to their corresponding control groups (CPACG associated with RP versus CPACG and APACG associated with RP versus APACG). Only 92 patients with CPACG and 84 patients with APACG were selected for the corresponding control groups. No significant differences were observed between the two groups with regard to ACD, AL, or RLP (all *P* > 0.05) (Table 1).

The mean ACD, LT, AL, and RLP values in the CPACG associated with RP group were 2.005 ± 0.257 mm, 4.905 ± 0.692 mm, 22.583 ± 0.949 mm, and 0.198 ± 0.021, respectively, and the mean ACD, LT, AL, and RLP values in the CPACG group were 2.044 ± 0.241 mm, 4.894 ± 0.413 mm, 22.353 ± 0.85 mm, and 0.201 ± 0.012, respectively. We observed no significant differences (*P* > 0.05) in any of the selected ocular biometric parameters between the two groups (Table 1). After controlling for the matching associations within each pair in the mixed linear regression model, we observed no significant differences (*P* > 0.05) between the two groups with respect to AL, ACD, and RLP (Table 2).

The mean ACD, LT, AL, and RLP values in the APACG associated with RP group were 1.673 ± 0.224 mm, 5.395 ± 0.39 mm, 22.112 ± 0.837 mm, and 0.198 ± 0.011, respectively, and the mean ACD, LT, AL, and RLP values in the APACG group were 1.729 ± 0.282 mm, 5.06 ± 0.385 mm, 21.983 ± 0.731 mm, and 0.194 ± 0. 014, respectively. With the exception of LT, none of the indicated ocular biometric parameters differed significantly between the two groups (*P* > 0.05; Table 3). After controlling for the matching associations within each pair in the mixed linear regression model, we noted no significant differences in AL, ACD, and RLP between the two groups (*P* > 0.05). The LT in patients with APACG associated with RP was significantly greater than that in patients with APACG.

## 5. Discussion

To the best of our knowledge, this is the first study to examine ocular biometric parameters in patients with PACG associated with RP and patients with PACG in a Chinese population. The association between PACG and RP has remained a controversial subject for many years. Some clinicians believe that cooccurrence of PACG and RP is not a coincidence, as the prevalence of PACG is higher in patients with RP than in patients without RP [9]. In addition, in clinical studies, patients with PACG associated with RP exhibited a shallower anterior chamber than patients with PACG after trabecular filtration surgery, which may indicate that the former group of patients possesses distinct biometric characteristics.

Previous reports have documented increases in the prevalence of glaucoma in patients with RP [21–24]. In our study, we used a more rigorous statistical method to minimize the statistical bias resulting from mismatches in age and gender. Patients with PACG associated with RP had almost the same biometric parameter characteristic as the patients with CPACG and APACG. Patients with APACG associated with RP had a significantly greater LT than patients with APACG (*P* < 0.05), a phenomenon that we attributed to measuring errors during the ultrasound examination. This significant difference between the groups was eliminated by RLP (*P* > 0.05). Duke Elder [9, 25] suggested that the association between PACG and RP may be coincidental. Although we observed no significant differences in most ocular biometry parameters between the two groups, we noted that patients with PACG associated with RP seemed to have a shallower ACD (2.005 ± 0.257 mm versus 2.044 ± 0.241 mm in the CPACG groups and 1.673 ± 0.224 mm versus 1.729 ± 0.282 mm in the APACG groups) and longer AL (22.583 ± 0.949 mm versus 22.353 ± 0.85 mm in the CPACG groups and 22.112 ± 0.837 mm versus 21.983 ± 0.731 mm in the APACG groups) than PACG patients.

In our study, the LT, as measured by A-scan, was inaccurate; thus, anterior segment optical coherence tomography (OCT) may be a more appropriate method of measuring LT. Additional studies regarding the relationship between PACG and RP are still needed, including studies assessing iris morphology and changes in anterior chamber volume in patients with PACG and RP.

In conclusion, patients with PACG associated with RP had the same biometric parameter characteristic as the patients with CPACG and APACG. This may suggest that RP has a coincidental relationship with angle-closure glaucoma. Additional studies are needed to correctly understand the differences in the anterior chamber structures of patients with these diseases.

## Figures and Tables

**Table 1:** Ocular biometry in the PACG associated with RP and PACG groups after propensity score matching.

	CPACG + RP	CPACG	*P* value	APACG + RP	APACG	*P* value
Number of eyes	23	92		21	84	
Male	10 (43.48%)	44 (47.83%)	0.817	9 (42.86%)	30 (35.71%)	0.617
Female	13 (56.52%)	48 (52.17%)		12 (57.14%)	54 (64.29%)	
Age	44.217 ± 14.107	49.207 ± 10.29	0.075	55.952 ± 10.509	55.738 ± 9.978	0.822
ACD	2.005 ± 0.257	2.044 ± 0.241	0.496	1.673 ± 0.224	1.729 ± 0.282	0.434
LT	4.905 ± 0.692	4.894 ± 0.413	0.946	5.395 ± 0.39	5.06 ± 0.385	0.001
AL	22.583 ± 0.949	22.353 ± 0.85	0.260	22.112 ± 0.837	21.983 ± 0.731	0.484
RLP	0.198 ± 0.021	0.201 ± 0.012	0.452	0.198 ± 0.011	0.194 ± 0.014	0.222

**Table 2:** Mixed linear regression model of ocular biometry in the CPACG associated with RP and CPACG groups.

	Regression coefficient	Standard error	*t* value	*P* value
ACD	−0.039	0.053	−0.727	0.469
LT	0.01	0.103	0.1	0.92
AL	0.231	0.196	1.176	0.243
RLP	−0.004	0.003	−1.138	0.258

**Table 3:** Mixed linear regression model of ocular biometry in the APACG associated with RP and APACG groups.

	Regression coefficient	Standard error	*t* value	*P* value
ACD	−0.056	0.066	−0.849	0.398
LT	0.335	0.084	3.984	0
AL	0.129	0.184	0.702	0.484
RLP	0.004	0.003	1.228	0.222

## Abbreviations

RP: Retinitis pigmentosa
PACG: Primary angle-closure glaucoma
UBM: Ultrasound biomicroscopy
ACD: Anterior chamber depth
LT: Lens thickness
AL: Axial length
CPACG: Chronic primary angle-closure glaucoma
APACG: Acute primary angle-closure glaucoma
RLP: Relative lens position
IOP: Intraocular pressure.

## Additional Points

*Availability of Data and Materials*. Please contact the authors for data requests.
